# Operating Endoscopically with “Two Hands” to Remove Calcified Permanent Suture After Pyeloplasty

**DOI:** 10.1089/cren.2015.0031

**Published:** 2016-01-01

**Authors:** Sean McAdams, Robert M. Sweet, James Kyle Anderson

**Affiliations:** Department of Urology, University of Minnesota, Minneapolis, Minnesota.

## Abstract

We describe a combined percutaneous and endoscopic approach to remove encrusted permanent suture in the renal pelvis that was placed during pyeloplasty repair. Our index patient had a laparoscopic dismembered pyeloplasty at an outside institution 10 years before presenting with flank pain and nondependent nephrolithiasis. This proved to be an encrusted permanent suture material. There is limited data on incidence of nephrolithiasis after ureteropelvic junction repair, but it is well documented that nonabsorbable suture lines should be avoided in the urinary tract as they may serve as a nidus for stone formation.

## Clinical History

**FIG. 1.**
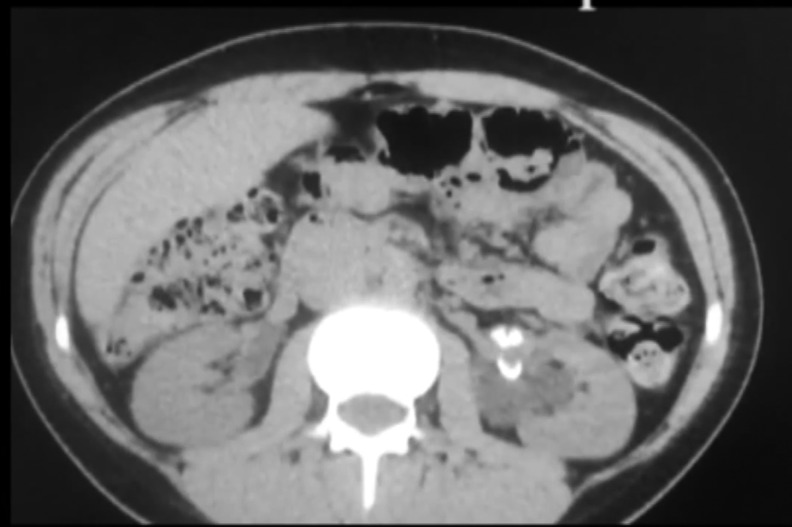
Noncontrast CT demonstrating nongravity-dependent calcifications at the *left* ureteropelvic junction with accompanying hydronephrosis.

A30-year-old female presented to us with 7 years of left flank discomfort that was worsening over the past several months. Her history was significant for left congenital ureteropelvic junction (UPJ) obstruction for which she underwent a laparoscopic dismembered pyeloplasty at an outside institution at age 20. Before our encounter, she underwent a CT scan that demonstrated left hydronephrosis and irregular calcifications at the left UPJ that were nongravity dependent (Fig. 1). The referring urologist did place a ureteral stent that provided relief of the patient's flank discomfort. She denies any history of urinary tract infections, hematuria, or kidney stones. She had no fevers or leukocytosis. Serum creatinine was normal and urine culture was negative.

## Physical Examination

The patient had normal vital signs. She appeared healthy. Mild left flank tenderness was present, otherwise her physical examination was nonremarkable.

## Intervention

**FIG. 2.**
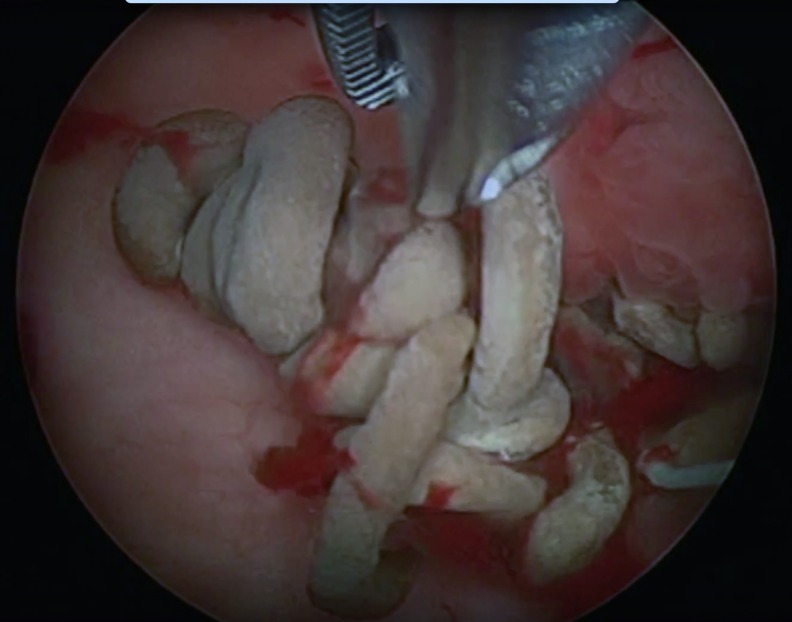
Appearance of calcified sutures in *left* renal pelvis.

**FIG. 3.**
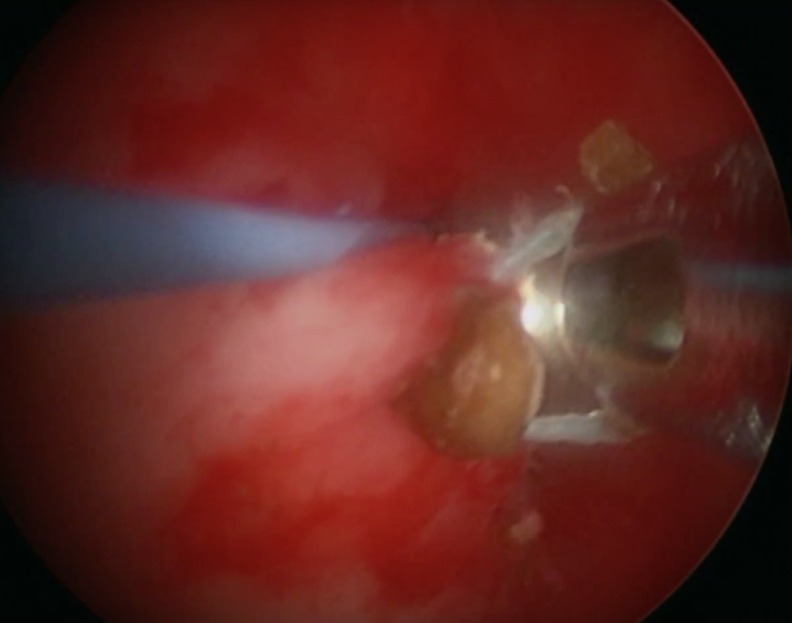
Application of tension on the suture material through nephroscopy enables ureteroscopic transection of the foreign body at the level of the urothelium and eliminates the foreign body from future urine exposure.

The split-leg prone position was used to allow dual access to the renal collecting system by using a nephroscope through the flank and ureteroscope through a ureteral access sheath.^1^ Ultrasonic lithotripsy (CyberWand, Olympus) was first used for reduction of stone burden and to provide exposure of suture material (Fig. 2). Two surgeons then worked in synchrony, using a grasping instrument through the nephroscope to apply tension to the permanent suture material and permit transection through holmium: YAG laser lithotripsy at the level of the urothelium (Fig. 3). A 200 μm laser fiber and laser settings of 0.3 Joules and 30 Hz were used for suture transection. Subsequently, a 500 μm laser fiber was used through the nephroscope to obliterate any residual protrusions of suture material from the urothelium. A Double-J 26 cm × 6F ureteral stent was left postoperatively. Operative time was 3 hours and blood loss was 100 mL.

## Results

The patient was visually stone free and all foreign suture material was removed from the renal pelvis. CT on postoperative day 1 was negative for residual stone and without abnormality. She was discharged the day after surgery and the ureteral stent was left in place for 2 weeks. Stone composition was 30% calcium oxalate and 70% calcium phosphate. The suture material was determined to be Ethibond, a nonabsorbable braided polyester suture. She was pain free at 6 months follow-up.

## Outcomes

Nonabsorbable suture should not be used for reconstruction in the urinary tract, including pyeloplasty surgery, because it may serve as a nidus for future stone formation. One should consider foreign body a possible etiology for stone formation in patients with nondependent renal pelvis stones and previous pyeloplasty surgery. Removal of all foreign suture material from the collecting system is desired to reduce likelihood of recurrent stone formation. Endoscopic suture removal in the renal pelvis can be performed safely and effectively by using percutaneous access and the split-leg-prone position. The combined percutaneous and endoscopic approach allows operation with “two hands” within the urinary tract so that suture can be cut while on tension.

## Abbreviations Used

CT: computed tomography
UPJ: ureteropelvic junction
